# Deletion of Mitochondrial Uncoupling Protein 2 Exacerbates Mitochondrial Damage in Mice Subjected to Cerebral Ischemia and Reperfusion Injury under both Normo- and Hyperglycemic Conditions

**DOI:** 10.7150/ijbs.48204

**Published:** 2020-08-25

**Authors:** Maotao He, Yanmei Ma, Rui Wang, Jianzhong Zhang, Li Jing, P. Andy Li

**Affiliations:** 1 Department of Pathology, General Hospital of Ningxia Medical University, Yinchuan, Ningxia 750004, China; 2 School of Basic Medical Sciences, Department of Pathology, Ningxia Medical University; Ningxia Key Laboratory of Vascular Injury and Repair, Yinchuan, Ningxia 750004, China; 3 Department of Pharmaceutical Sciences, Biomanufacturing Research Institute and Technological Enterprise (BRITE), College of Health and Sciences, North Carolina Central University, Durham, NC 27707, USA

**Keywords:** Uncoupling protein 2, cerebral ischemia, hyperglycemia, mitochondrial dynamics, mitochondrial fission, mitochondrial ultrastructure, ROS.

## Abstract

Deletion of mitochondrial uncoupling protein 2 (UCP2) has been shown to aggravate ischemic damage in the brain. However, the underlying mechanisms are not fully understood. The objective of this study is to explore the impact of homozygous UCP2 deletion (UCP2^-/-^) on mitochondrial fission and fusion dynamic balance in ischemic mice under normo- and hyperglycemic conditions. UCP2^-/-^ and wildtype mice were subjected to a 60 min middle cerebral artery occlusion (MCAO) and allowed reperfusion for 6h, 24h and 72h. Our results demonstrated that deletion of UCP2 enlarged infarct volumes and increased numbers of cell death in both normo- and hyperglycemic ischemic mice compared with their wildtype counterparts subjected to the same duration of ischemia and reperfusion. The detrimental effects of UCP deletion were associated with increased ROS production, elevated mitochondrial fission markers Drp1 and Fis1 and suppressed fusion markers Opa1 and Mfn2 in UCP2^-/-^ mice. Electron microscopic study demonstrated a marked mitochondrial swolling after 6h of reperfusion in UCP2^-/-^ mice, contrasting to a mild mitochondrial swolling in wildtype ischemic animals. It is concluded that the exacerbating effects of UCP2^-/-^ on ischemic outcome in both normo- and hyperglycemic animals are associated with increased ROS production, disturbed mitochondrial dynamic balance towards fission and early damage to mitochondrial ultrastructure.

## Introduction

Uncoupling protein 2 (UCP2) is a member of inner mitochondrial membrane proteins, that dissipates the mitochondrial proton gradient by transporting H^+^ across the inner membrane, thereby generating heat, stabilizing the inner mitochondrial membrane potential and reducing the formation of reactive oxygen species (ROS) 1,2. Several studies have suggested UCP2 plays a vital role in the pathological process of neural damage after cerebral ischemia and reperfusion (I/R) 3, 4, 5. Our previous studies have shown that deletion of the UCP2 gene exacerbates ischemic infarct volume, upregulates the protein levels of the inflammatory cytokines, and suppresses antioxidant, cell-cycle, and DNA repair genes in normoglycemic animals 6. And that overexpression of UCP2 inhibits pro-inflammatory cytokines and activates cell cycle and cell survival factors 7.

Many studies have identified diabetes mellitus as an independent and significant risk factor for stroke as well as stroke-related mortality 8, 9, 10. Both experimental and clinical studies have shown that hyperglycemia, one of the main characteristics of diabetes mellitus, further exacerbates ischemia/reperfusion, activating cell survival pathways 11, 12, causing early damage to astrocytes and mitochondria, and inhibiting mTOR and ERK1/2 signaling 13, 14, 15. However, the role of UCP2 in hyperglycemic ischemic damage has not been studied. In this study, we examined the effects of UCP2 deletion on ischemic outcomes in normo- and hyperglycemic mice subjected to a transient middle cerebral artery occlusion (MCAO).

Mitochondrial dynamics refers to the balance of fusion and fission in the mitochondrial network to maintain their shape, distribution, and size. Mitochondrial fission is ensured by dynamin-related protein 1 (Drp1) and fission 1 (Fis1), while Mitofusins 1, 2 (Mfn1, Mfn2) and optic atrophy 1 (Opa1) mediate mitochondrial fusion process 16. Mitochondrial dynamics is closely associated with mitochondrial function and neurons are particularly sensitive to perturbations in mitochondrial dynamics. Accumulative evidence has revealed a close link between imbalanced mitochondrial dynamics and neurodegenerative diseases. Recent reports have suggested that mitochondrial fission is an early event required for ischemic neuronal death 17. Mitochondrial fission occurs as early as 3 h after reperfusion in ischemic mice 18. In contrast, loss of mitochondrial fusion protein Mfn2 contributes to enhanced ischemia/reperfusion injury 19. In both in vivo and in vitro ischemic models, the expression of Mfn2 is decreased, which leads to mitochondrial dynamics imbalance and disruption of Ca^2+^ homeostasis 20. Hyperglycemia further tilts the mitochondrial dynamic imbalance toward fission by increasing the levels of fission markers and decreasing fusion proteins in the early reperfusion stage 21. However, it is unknown whether UCP2 regulates mitochondrial fission and fusion in the setting of cerebral I/R injury under normo- and hyperglycemic conditions. The objective of this study was to explore the impact of UCP2 deletion on mitochondrial dynamic balance in ischemia and reperfusion injury under both normo-and hyperglycemic conditions.

## Materials and Methods

### Materials

Streptozotocin (STZ) and 2,3,5-Triphenyl Tetrazolium Chloride (TTC) were obtained from Sigma. A Reactive Oxygen Species Assay Kit was purchased from Beyotime (Jiangsu, China). TUNEL Assay Kit was obtained from Roche (Mannheim, Germany). Opa1 (ab90857) and Mfn2 (ab50843) antibodies were purchased from Abcam. Fis1 (PA5-22142) antibody was purchased from Thermo Fisher scientific. Drp1 (#8570) antibody was purchased from Cell Signaling Technology and anti-β-actin was purchased from Bios (Beijing, China).

### Animals and groups

A total of 286 male UCP2^-/-^ mice and wildtype (WT) mice were used in this study. All animal procedures were performed following the NIH Guide for Care and Use of Laboratory Animals and were approved by the Institutional Animal Care and Use Committee at Ningxia Medical University. Breeding pairs of UCP2^-/-^ mice were obtained from the Jackson laboratory and their off-springs were genotyped. The mice were maintained in a specific pathogen-free colony of the Laboratory Animal Center of Ningxia Medical University (Yinchuan, China) with controlled temperature, humidity, and 12:12 hour light and dark cycle.

UCP2^-/-^ mice and WT mice were randomly divided into normo- and hyperglycemic groups, each consisting of four subgroups: a sham-operated control and 1h MCAO plus 6h, 24h, and 72h of reperfusion. The animal groups and numbers used in each group are summarized in Table 1. Hyperglycemia was induced by intraperitoneal injection of STZ (120 mg/kg) that was freshly dissolved in 0.1 M citrate buffered saline (pH 4.5). Age-matched mice receiving the same volume of 0.1 M citrate-buffered saline served as normoglycemic controls. The blood glucose level was measured after 72h of STZ injection using Blood Glucose Meter (Boshilong, Taiwan). Animals with a blood glucose level higher than 16.8 mmol/L were designated as the hyperglycemia mice based on our previous experience 22.

### Ischemia and reperfusion model

The animals were anesthetized with 3% isoflurane for induction and maintained at 1.0 - 1.5% during the surgical procedures. The anesthesia was delivered in a 70% nitrous oxide and 30% oxygen mixture using a facemask (MATRX VIP 3000). Cerebral ischemia was induced by middle cerebral artery occlusion (MCAO). Briefly, the internal carotid artery (ICA), external carotid artery (ECA), and the common carotid artery (CCA) were isolated through a midline incision. The right CCA was ligated, and the right ICA was temporarily closed by a loose suture. A small incision was made on the CCA and a filament (Doccol corporation, USA), which had a distal cylinder of silicon rubber with a diameter of 0.21±0.02 mm, was inserted into the ICA until a faint resistance was felt. After 60 min occlusion, the filament was withdrawn to achieve recirculation. The sham animals were subjected to the same surgical procedure as the MCAO mice but without occlusion of the MCA. During the surgery period, the body temperature of the mice was maintained with a heating pad and lamp and monitored by a rectal thermometer (Omron, Dalian, China). The mice were subjected to a neurological examination immediately after the animals recovered from anesthesia to judge the successful induction of MCAO model and again after 24h reperfusion to compare the functional recovery between the experiential groups. The neurological deficit was scored by Zea-Longa scale: 0, no neurological deficits; 1, failure to fully extend left paw; 2, circling to the left; 3, falling to the left; 4, unable to walk spontaneously and exhibiting depressed levels of consciousness. The animal with scores of 2 and above was selected as the successful MCAO model. Five animals were excluded due to lack of neurological deficit after MCAO. All animals were coded with a number and the people who further process the measurements and analysis were blinded to the experimental conditions.

### Anatomy of the MCA and Circle of Willis

The animals were deeply anesthetized and transcardially perfused with 0.9% saline to flush out the blood. Mouse was injected with Indian ink (2%) made in 20% gelatin and saline. After perfusion brain was cooled to allow gelatin solidification and fixed with 4% paraformaldehyde. Brain images were captured using a Nikon digital camera.

### Measurement of infarct volume and edema

The mice were sacrificed at 24 h after MCAO and whole brains were dissected coronally into 1-mm brain slices using a stainless brain matrix (68707, RWD, Shenzhen). The brain slices were immediately placed into a 24-well plate and incubated with 2, 3, 5-triphenyltetrazolium chloride (TTC, 2%) at 37 °C for 15 min and then fixed in 4% paraformaldehyde. TTC stains viable brain tissue as deep red but infarcted tissues as pale color. Areas of infarcted tissue were measured using NIH Image J software (rsb.info.nih.gov/nih-image) and infarct volumes were calculated from all sections with corrections of intersection distance. The infarcted volume was expressed as the percentage of infarcted tissue relative to total brain tissue. Edema volume was semi-quantitatively analyzed after 24 h reperfusion. The relative edema volume (%) was calculated as: (ipsilateral hemisphere volume - contralateral hemisphere volume) / (contralateral hemisphere volume) ×100%.

### ROS detection

Frozen mice brain sections were cultured and incubated in the dark with 10 μM/L of Dihydroethidium (DHE) for 30 min at 37°C. Intracellular ROS production was assessed with an Olympus FluoView1000 Laser Scanning Confocal Microscope (using ex/em λ=480 nm/535 nm for DHE).

### TUNEL staining

Terminal deoxynucleotidyl transferase mediated dUTP nick‑end labeling (TUNEL) staining was used to detect apoptosis cells (Roche, #11684795910) according to the manufacturer's protocol. The number of TUNEL-positive cells was counted in five microscopic fields at 400 X.

### Immunohistochemistry assay

Paraformaldehyde-fixed and paraffin-embedded brain tissues were sectioned (4 µm thickness). The sections were submerged in citrate buffer (pH = 6.0) and heated at boiling temperature under a high-pressure situation for 5 min for antigen retrieval. The expression levels of Fis1 (1:400, PA5-22142, Thermo Fisher), Opa1(1:200, ab90857, Abcam), Drp1(1:200, #8570, CST) and Mfn2 (1:200, ab50843, Abcam) proteins were examined in each group after primary antibody incubation overnight and secondary antibody incubation at 37°C for 45 min. The reaction was visualized with DAB (ZSGB-BIO, Beijing, China) staining and then hematoxylin counterstaining the nuclei. The number of positively stained cells was counted in five microscopic fields at 400 X.

### Western blot

The brain tissues were homogenized on ice with lysis buffer. We determined the protein concentration of each protein sample to ensure that the sample volumes were consistent. Equal amounts (50 µg) of protein extracts were subjected to 10%-12% sodium dodecyl sulfate-polyacrylamide gels electrophoresis (SDS-PAGE) and transferred to polyvinylidene fluoride membranes (Millipore). The membranes were incubated overnight at 4°C with the following primary antibodies: anti-Fis1 (1:1000), anti-Opa1 (1:1000), anti-Mfn2 (1:1000) and anti-Drp1 (1:1000). Then, the membranes were incubated with secondary antibodies for 1h at room temperature. Imaging was performed using the BIO-RAD Imaging System with chemiluminescence detection reagents. Semi-quantitative results were obtained by measuring the optical density of the target bands and were expressed as the ratio of each targeted protein to β-actin. We make the band densities in WT+NG sham groups as 100 to compare differences in other group. Band relative densities were analyzed by NIH Image J software (rsb.info.nih.gov/nih-image).

### Electron Microscopic Studies

Brains were perfusion fixed with 2% glutaraldehyde at 6 h of reperfusion collected from both normoglycemic and hyperglycemic animals. The brain sections were taken between Bregma 1 to -1 mm and post-fixed with 4% glutaraldehyde in 0.1 mol/L cacodylate buffer (pH 7.4). The sections were then soaked in 1% osmium tetroxide in 0.1 M cacodylate buffers for 2 h and stained with 1% aqueous uranyl acetate overnight. Tissue sections were dehydrated in ascending series of ethanol to 100% followed by dry acetone and embedded in epoxy resin. Ultrathin sections were cut and counterstained with lead citrate before examination by transmission electron microscope (H7650).

### Statistics

All data are presented as means ± SD. Statistical analysis was performed using one-way ANOVA with SPSS 19.00. Student's t-test was used to analyze the difference in infarct volume between the two animal species. Statistical significance was determined as *p* < 0.05. The numbers of animals in each subgroup are given in Table 1 and in Figure legends.

## Results

### Cerebral vasculature and blood glucose levels in transgenic and WT mice

**Figure 1:**
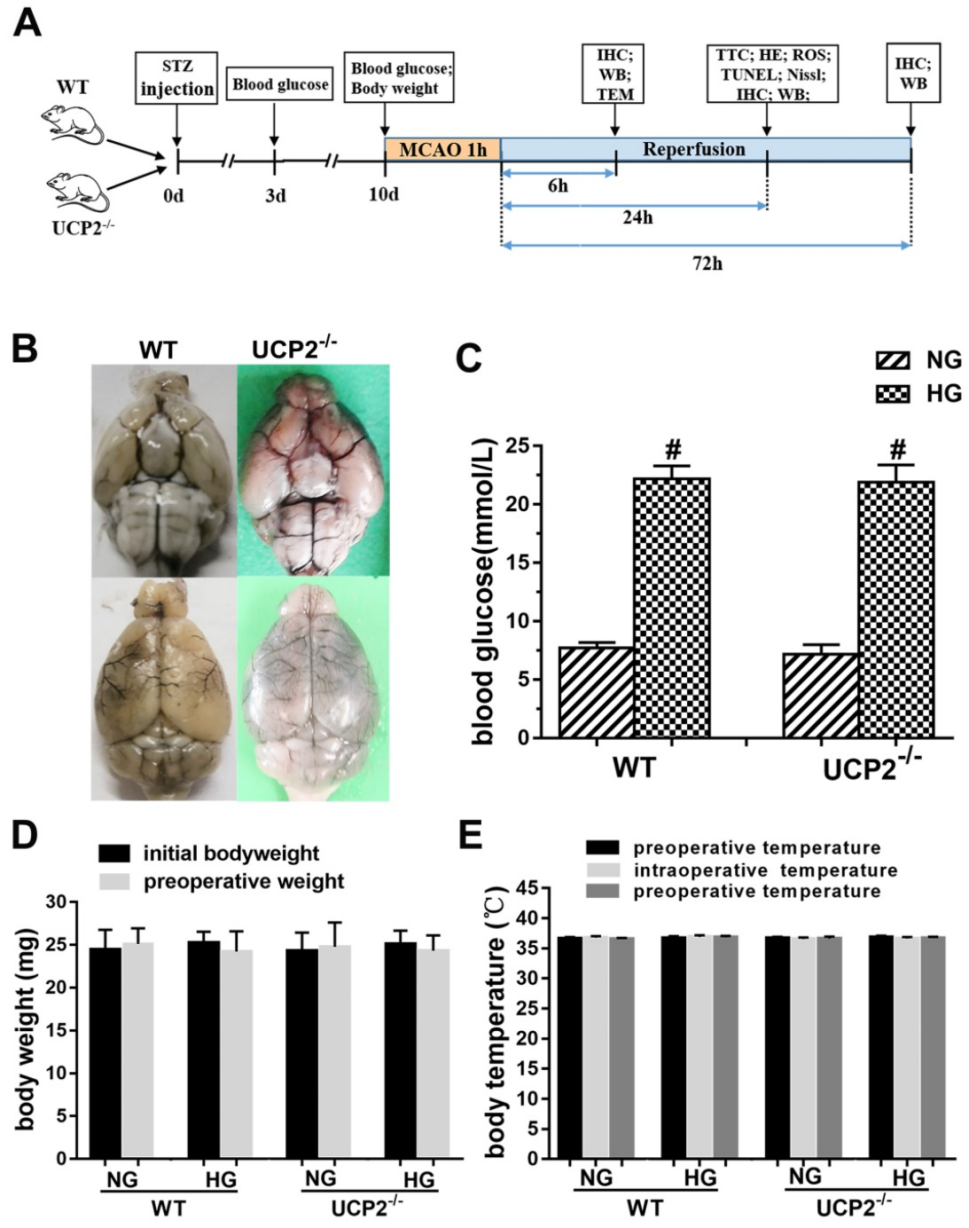
** Effect of UCP2 deletion on the onset of high glucose in different mice.** (A) This diagram illustrated the experimental design. (B) Cerebral vasculature. Mice (n = 3, each group) were perfused with Indian black ink to determine whether there were vascular abnormalities in the UCP2^-/-^ mice. The Circle of Willis, anterior cerebral arteries, middle cerebral arteries, and posterior arteries all appear normal as compared with those in WT controls. (C) Blood glucose level. The blood glucose was obviously higher in the STZ induced diabetic mice than those in control group (*p*<0.01). There was no significant in WT mice and UCP2^-/-^ mice (n = 54, each group). (D) Body weight. There was no significant different between initial and preoperative weight in different groups (n = 54, each group). (E) Body temperature. There was no significant among preoperative temperature, intraoperative temperature and postoperative (n = 54, each group).

The experimental protocol is illustrated in Fig. 1A. To evaluate whether UCP2 deletion causes phenotypic changes in the cerebral vasculature, we transcardially injected Indian black ink and imaged the cerebral blood vessels (Fig. 1B). The result showed intact and correct alignment of the circle of Willis, anterior, middle, and posterior cerebral arteries with no remarkable difference between WT and UCP2^-/-^ mice. Physiological variables were measured and maintained constant. Body temperature was kept at 36.5°C to 37.5°C. As expected, the blood glucose (Fig. 1C) was significantly higher in the STZ induced hyperglycemia mice than those in control group (*p*<0.01). The average blood glucose level in the WT normal glucose group (WT+NG) was 7.70±1.32 mM, while that in the WT hyperglycemic group (WT+HG) was 22.19±6.10 mM (*p*<0.01 vs. WT+NG). Similarly, blood glucose concentration was 7.15±2.00 mM in UCP2^-/-^ normal glucose group (UCP2^-/-^+NG) and 21.91±6.31 mM in UCP2^-/-^ hyperglycemic group (UCP2^-/-^+HG) (*p*<0.01 vs. UCP2^-/-^ +NG). However, there was no difference in blood glucose level between WT and UCP2^-/-^ animals. Animal body weight and body temperature were the same between UCP2^-/-^ and WT under same glycemic conditions (Fig. 1D and 1E).

### UCP2 deletion aggravated ischemic brain damage in both normo- and hyperglycemic mice

**Figure 2:**
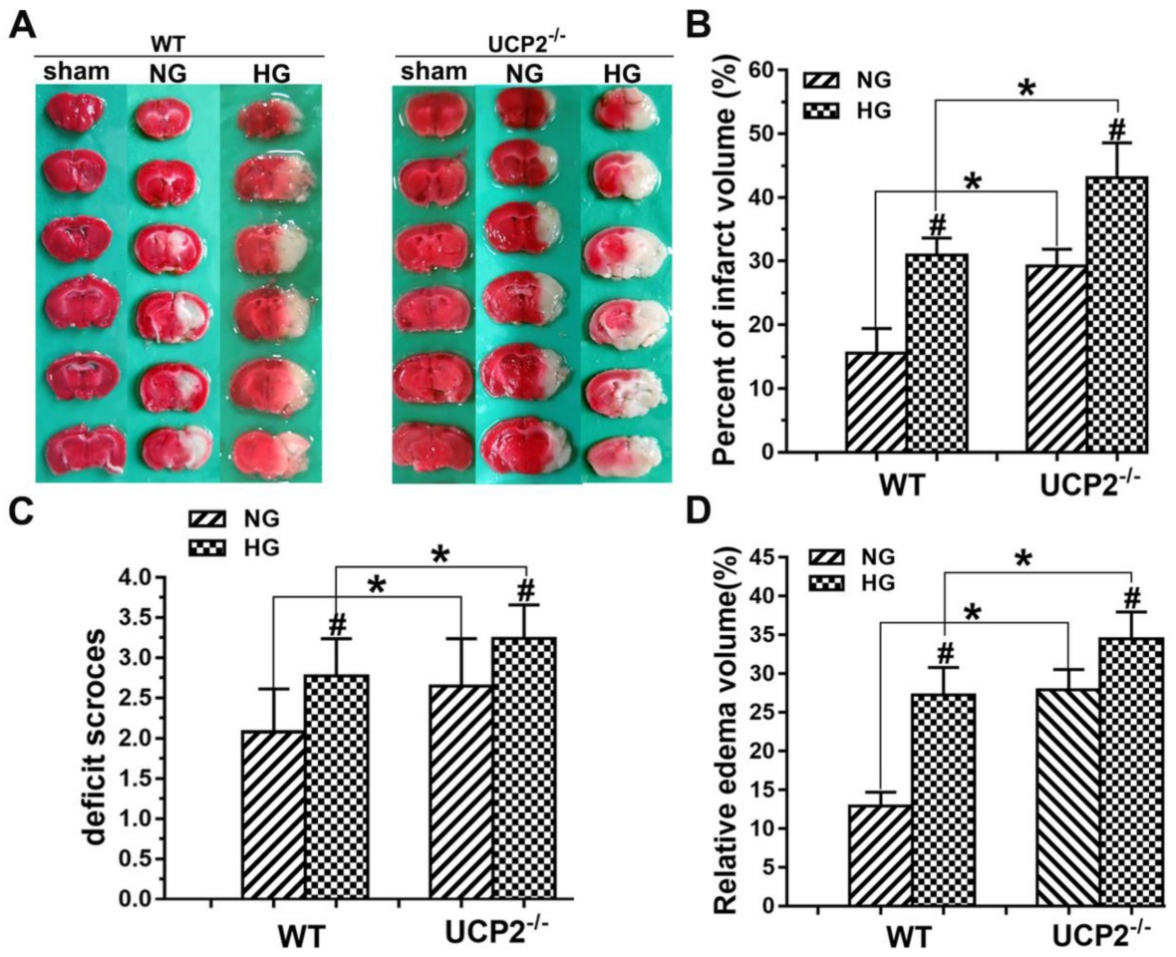
** UCP2 deletion aggravated ischemic brain damage in hyperglycemic mice.** (A) Representative TTC stained brain sections showing infarct volume (white color) at 24-h of reperfusion following 60min of MCAO in WT and UCP2^-/-^ mice. There were 4 animals for WT each group and 5 animals for UCP2^-/-^ each group. (B) Bar graph summarizes the mean values of cerebral infarction in WT and UCP2^-/-^ mice. Infarct volume enlarged significantly in UCP2^-/-^ mice with high glucose (n = 4 in each WT group; n = 4 in each UCP2^-/-^ group).^**#**^*p*<0.05 *vs*. NG; ^*^p<0.05 *vs*. WT. (C) Assessments of neurological deficits (n=20 in each group). **^#^***p*<0.05 *vs.* NG; ^*^p<0.05 *vs.* WT. (D) Quantitative analysis of edema volume. There were 4 animals for WT each group and 5 animals for UCP2^-/-^ each group. **^#^***p*<0.05 *vs.* NG; ^*^p<0.05 *vs.* WT, respectively.

To determine whether the deletion of UCP2 can aggravate ischemic brain damage under both normo- and hyperglycemic conditions, we examined infarct volumes and neurological deficits. First, the results showed that UCP2 deletion increased infarct volume compared to WT mice under normoglycemic condition. Focal ischemia of 60 min duration induced brain infarct in the striatum, without much involvement of the overlaying cortex in WT mice at 24h of reperfusion. The infarct volume was significantly enlarged in UCP2^-/-^ mice compared with the WT mice, which covered both the striatum and the cortex. Second, hyperglycemia enlarged infarct volume compared with that in normoglycemic WT mice (Fig. 2A and 2B). Third, UCP2 deletion with hyperglycemia further expanded the infarct volume compared with WT mice under hyperglycemic condition. Similarly, UCP2^-/-^ mice showed a significant increase in neurological deficit scores compared with WT ischemic mice (Fig. 2C), indicating neurological deficit scores were positively correlated to the infarct size. Compared with the WT+HG group, UCP2^-/-^ +HG group had more severe neurological deficit scores after ischemia/reperfusion (*p*<0.05). Measurement of brain edema also suggested that UCP2^-/-^ significantly increased brain edema compared to WT mice under both normo- and hyperglycemic ischemic conditions (*p*<0.05, Fig. 2D). Our data demonstrate that UCP2 deletion aggravated ischemic brain damage in both normo- and hyperglycemic mice.

### UCP2 deletion aggravated histopathological changes and apoptotic death after ischemia under both normo- and hyperglycemic conditions

**Figure 3:**
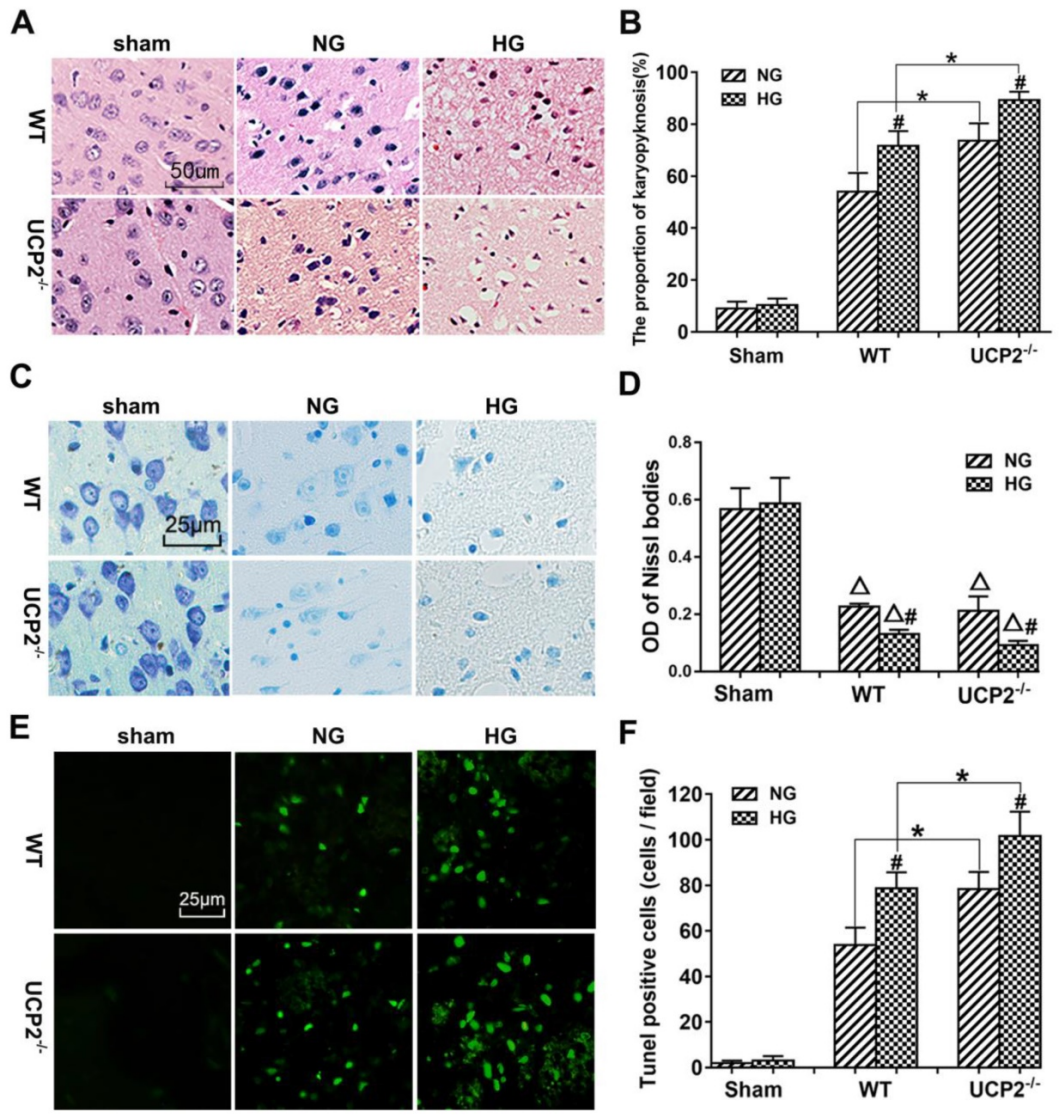
** UCP2 deletion aggravated ischemia/reperfusion induced histopathological changes and apoptotic in hyperglycemic mice.** (A) HE staining. (B) Quantitative summary of pyknotic cells. (C) Nissl staining. (D) The average optical density of each group of Nissl bodies. (E) Apoptosis was determined via TUNEL test. (F) the number of TUNEL positive cells (n=6 mice, each group). ^Δ^*p*<0.05 *vs.* sham group;^*^p<0.05* vs.* WT group; ^#^*p*<0.05 *vs.* NG group, respectively.

The pathological outcomes in the cortex are given in Figure 3A and 3B. As shown in Figure 3A, a few scattered dead neurons were observed in the sham-operated animals. Transient cerebral ischemia resulted in a mildly increased number of dead neurons in the cortex after 24h of reperfusion in WT mice (*p*<0.01). Deletion of UCP2 further increased the percentage of dead neurons in the cortex 24h after reperfusion compared with the WT counterpart under both euglycemic and hyperglycemic conditions (Fig. 3A and 3B). As expected, hyperglycemia caused more ischemic cell death than normoglycemic animals in both WT and UCP2^-/-^ mice. Interestingly, Nissl staining showed a significant decrease after ischemia in WT animals with euglycemic condition. Deletion of UCP2 did not seem to further reduce to the Nissl staining in euglycemic mice. However, deletion of UCP2 in hyperglycemic animals significantly decreased Nissl body's density compared with WT mice in hyperglycemic group (Fig. 3C and 3D). TUNEL staining images were taken from the ischemic penumbra area in the ipsilateral cortex. We selected the penumbral area because most apoptotic cells present in this area. Our results revealed ischemia increased the number of TUNEL positive cells. Similarly, hyperglycemic ischemia increased the number of TUNEL positive cells compared with normoglycemic ischemia and UCP2 deletion further elevated the numbers of TUNEL positive cells after ischemia in both normo-and hyperglycemic animals compared with the WT counterparts (Fig. 3E and 3F). The right corner of the WT+HG image was taken close to the ischemic core. There was less TUNEL positive neurons in this area probably due to the fact that severe insult in HG ischemia led to more necrotic, instead of apoptotic, cell death.

### UCP2 deletion enhanced ROS production in both normo- and hyperglycemic mice after ischemia

**Figure 4:**
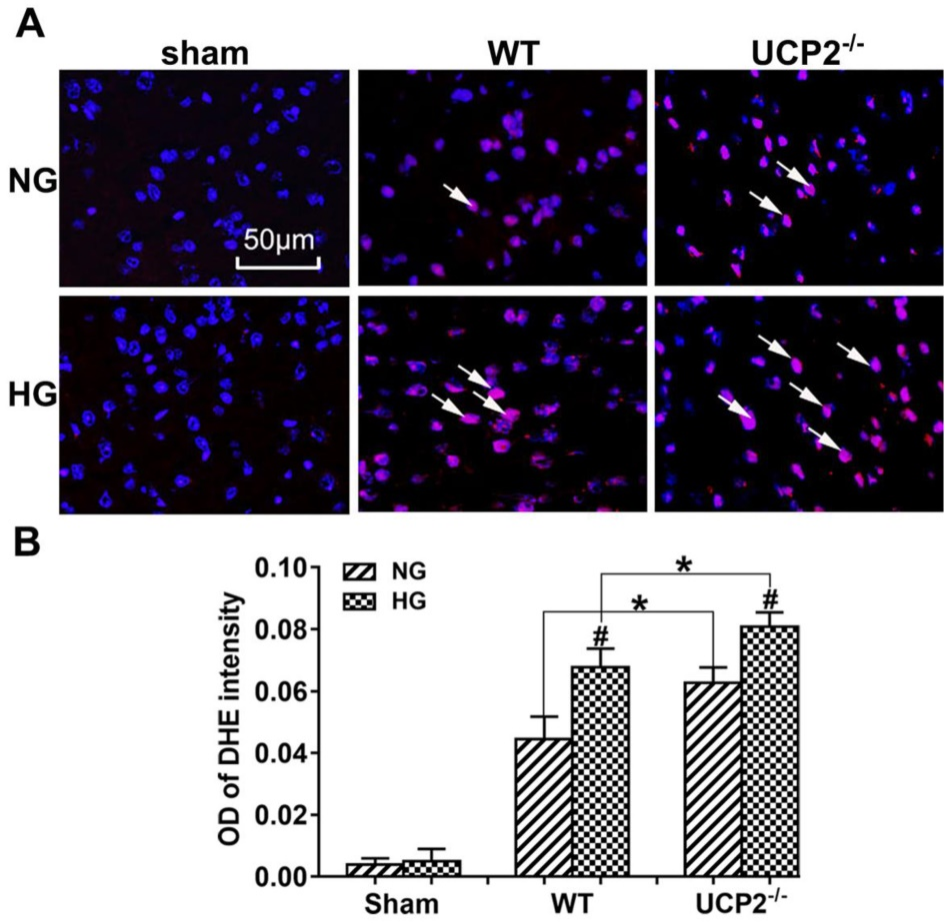
** UCP2 deletion enhanced ROS production in hyperglycemic mice.** (A) ROS production detected by DHE in WT and UCP2^-/-^ mice. Nuclei were labeled with DAPI. Magnification, 400X. Scale bar= 50 μm. (B) summarized DHE fluorescent intensity. *n*=4 mice per group. Data are presented as means ± SD. ^*^p < 0.001 *vs.* WT group in same glucose and *^#^p* < 0.05 *vs.* NG group in some type of animal.

As shown in Figure 4A and 4B, MCAO induction caused a significant enhancement of superoxide production as detected by DHE. Consistently, hyperglycemia further increased the ROS compared with normoglycemic mice after ischemia and reperfusion at 24h in the cortex and deletion of UCP2 led to a much more pronounced elevation of ROS in both normo- and hyperglycemic animals after ischemia and reperfusion injury compared with their matched WT counterparts.

### UCP2 deletion further exacerbated the ischemia-induced mitochondrial dynamic imbalance

**Figure 5:**
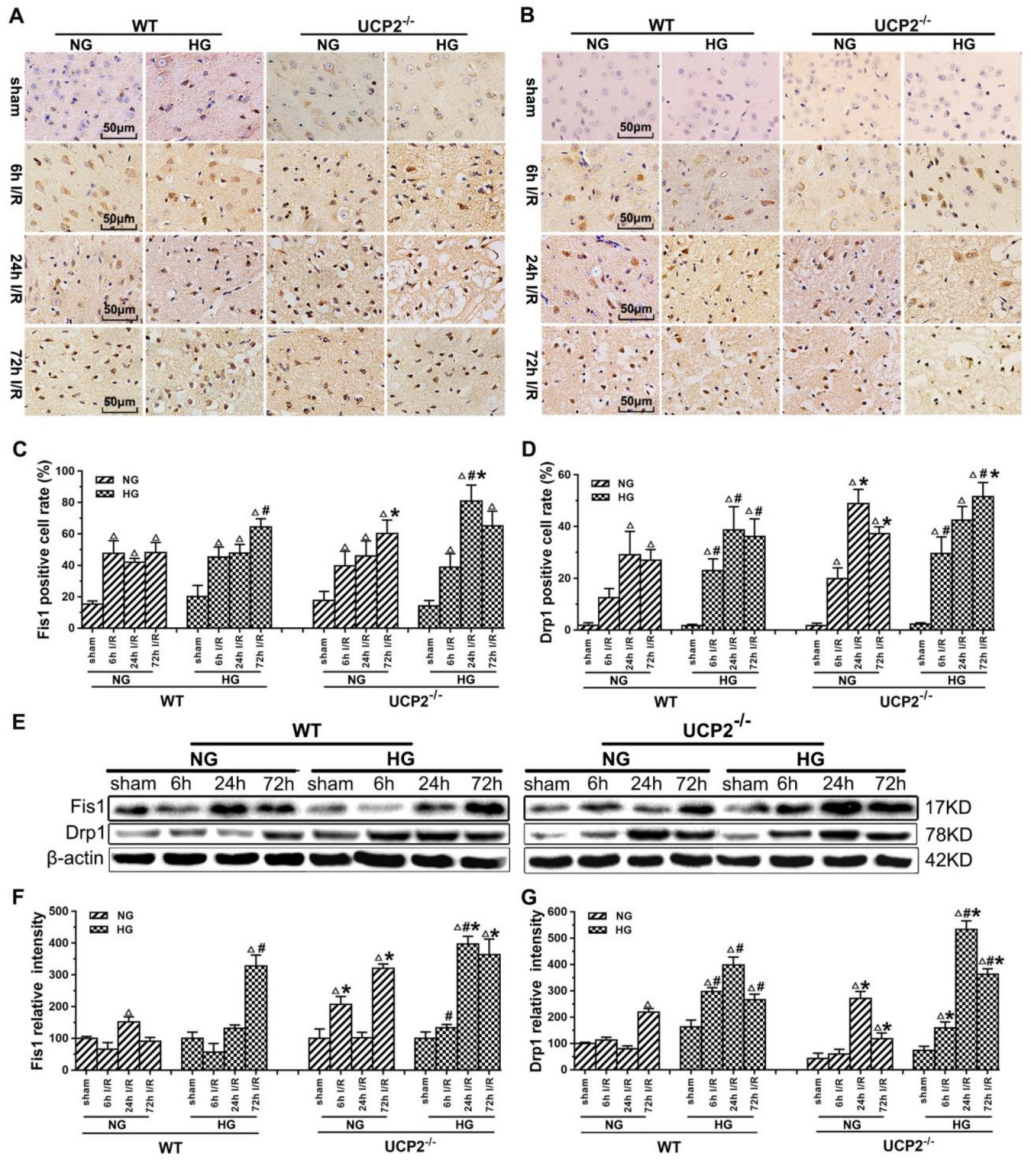
** UCP2 deletion increased the levels of mitochondrial fission-related proteins in hyperglycemic mice after ischemia/reperfusion.** Detections of mitochondrial fission-related proteins by immunohistochemistry and Western blotting (n=6, per group). (A, B) Representative photomicrographs for Fis1 and Drp1. Scale bar=50 μm. (C, D) Quantification of Fis1 and Drp1 immunointensity. E: Representative Western blots for Fis1 and Drp1. (F, G) Semi-quantification of Fis1 and Drp1 protein bands. Data are shown as mean ± SD. For values in F and G, the values in WT+NG sham group were converted to 100 and percent changes were presented for other groups relative to the WT+NG sham. ^Δ^*p*<0.05 *vs.* respective sham-operated controls, ^#^*p*<0.05 *vs.* normoglycemic and *p<0.05 *vs.* WT counterpart at an identical reperfusion stage.

**Figure 6:**
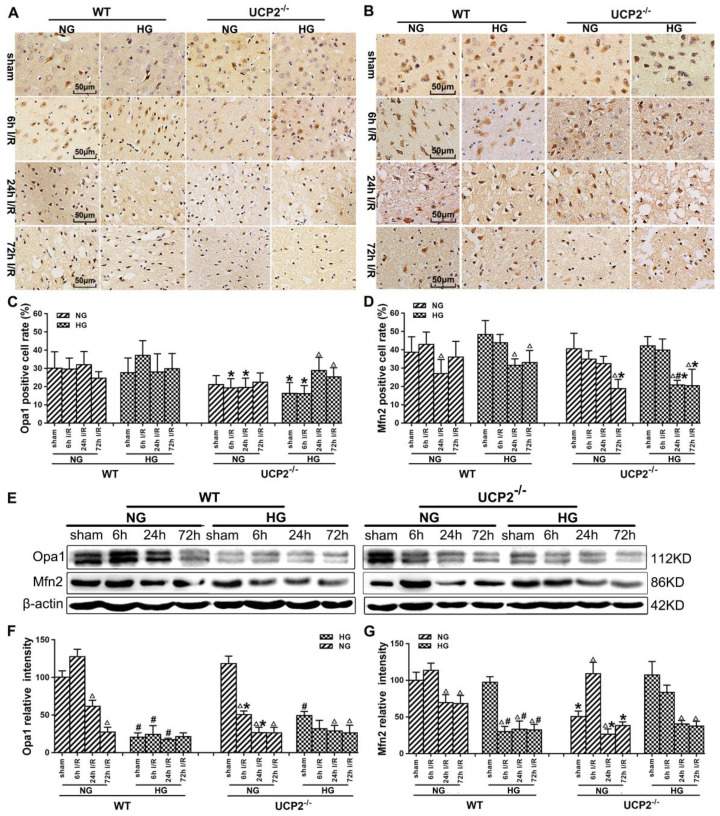
** UCP2 deletion increased the levels of mitochondrial fusion-related proteins in hyperglycemic mice after ischemia/reperfusion.** Detections of mitochondrial fusion-related proteins by immunohistochemistry and Western blotting. (A, B) Representative photomicrographs for Mfn2 and OPA1. Scale bar=50 μm. (C, D) Quantification of Mfn2 and OPA1 immunointensity. E: Representative Western blots for Mfn2 and OPA1. (F, G) Semi-quantification of Mfn2 and OPA1 protein bands.* n*=6 mice per group. Data are shown as mean ± SD. For values in F and G, the values in WT+NG sham group were converted to 100 and percent changes were presented for other groups relative to the WT+NG sham. ^Δ^*p*<0.05 *vs.* respective sham-operated controls, ^#^*p*<0.05 *vs.* normoglycemic and *p<0.05 *vs.* WT counterpart at an identical reperfusion stage.

Mitochondrial dynamics constantly change between fission and fusion status. To understand the mechanism by which UCP2 modulates mitochondrial fission/fusion balance, we detected mitochondrial fission and fusion-associated proteins in cortical samples collected from animals subjected to 60 min of ischemia and followed by 6h, 24h and 72h of reperfusion by immunohistochemistry and Western blot (Fig. 5 and Fig. 6).

The results of immunohistochemistry for Fis1 and Drp1 are given in Figure 5A-5D. The positive cells were labeled in brownish yellow color, and hematoxylin counterstaining stained the nuclei in blue. The expression of mitochondrial fission-related proteins was evaluated by the ratio of the number of positive cells to the total number of cells. Ischemia in normoglycemic WT mice induced a significant elevation of Fis1 and its level already peaked at 6h of reperfusion and maintained at that high level up to 72h (Fig. 5A and 5C). UCP2 deletion caused a further elevation of Fis1 in both normo- and hyperglycemic mice after ischemia compared with the WT mice. Changes of Drp1 were similar to those of Fis1. Thus, UCP2 deletion further pushed the Drp1 to higher levels at 24h and 72h in normoglycemic and 72h in hyperglycemic animals than in the WT counter groups (Fig. 5B and 5D). These results were further supported by Western blot. As shown in Figure 5E-5G, I/R injury upregulated the expression of proteins related to mitochondrial fission such as Fis1 and Drp1 over reperfusion time. The trend of Fis1 changes was similar to the results of immunohistochemistry (Fig. 5E and 5F). However, the level of Drp1 reached a peak at 24h in UCP2^-/-^ mice and was significantly higher than that in WT mice in hyperglycemic group (Fig. 5E and 5G).

Immunohistochemistry detection of mitochondrial fusion proteins Opa1 and Mfn2 revealed decreases of these two proteins in UCP2^-/-^ mice (Fig. 6A-6D). As shown, Opa1 level decreased slightly in WT mice after 72h of reperfusion, while UCP2 deletion decreased Opa1 content at 6h and 24h compared with WT ischemic mice (Fig. 6A and 6C). UCP2 deletion in hyperglycemic animals lowered Opa1 values in control and at 6h of reperfusion. UCP2 deletion resulted in a significant reduction of Mfn2 at 72h of reperfusion in normoglycemic ischemic animals and at 24h and 72h in hyperglycemic ischemic animals (Fig. 6B and 6D), suggesting that deletion of UCP2 suppressed mitochondrial fusion. Western blot results showed that ischemia in WT suppressed Opa1 at 24h and 72h of reperfusion (Fig. 6E-6G). The deletion of UCP2 in normoglycemic mice led to an early reduction of Opa1 in normoglycemic animals (Fig. 6E and 6F). Hyperglycemia resulted in a more pronounced decline of Opa1in WT than in normoglycemic group in all endpoints including non-ischemic control; however, it did not further decrease Opa1 in UCP2^-/-^ mice. Opa1 level in hyperglycemic animals was lower than that of the normoglycemic animals as revealed by two-way ANOVA analysis (*p*<0.05). The protein level of Mfn2 in the mitochondrial fraction decreased after cerebral ischemia and reperfusion in normoglycemic animals (Fig.6E and 6G). Overall, the Opa1 and Mfn2 levels were intended to be lower in hyperglycemic ischemia than in normoglycemic ischemia and deletion of UCP2 affected these fusion markers in normoglycemic ischemic mice.

**Figure 7:**
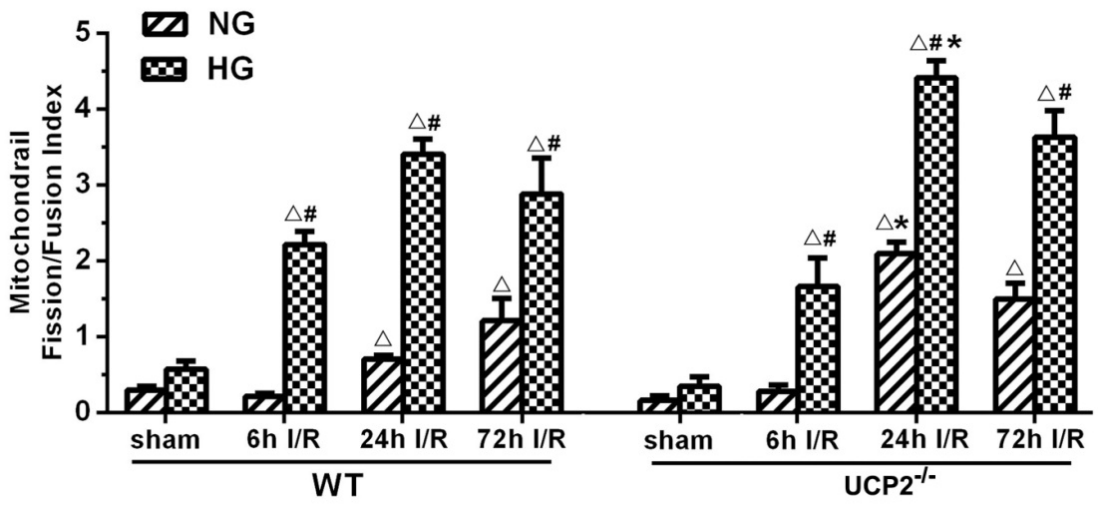
** Mitochondrial fission/fusion index following cerebral ischemia and reperfusion under both normo- and hyperglycemic conditions.** Index represents the ratio of fission (Drp1 and Fis1) and fusion (Opa1 and Mfn2) proteins. Data are expressed as means ± SD. △*p*<0.05, vs. respective sham-operated controls, ^#^*p*<0.05 vs. normoglycemic and *p<0.05 vs. WT counterpart at an identical reperfusion stage.

To better distinguish the effects of UCP2 knockout on the dynamic balance of mitochondrial fission and fusion, we summarized the levels of mitochondrial fission and fusion markers, calculated the ratio of fission and fusion, and expressed the data as the mitochondrial fission/fusion index. As shown in Figure 7, the fission/fusion index began to rise and peaked at 24h in UCP2^-/-^ mice, while mitochondrial fission/fusion index began to rise at 24h and reached its peak at 72h in WT mice under normoglycemic condition. The finding indicates that in normoglycemic animals, deletion of UCP2 induced mitochondrial fission/fusion index to reach the peak early and is significantly higher than the WT group. In the hyperglycemic group, mitochondrial fission/fusion index began to increase at 6h and peaked at 24h. The ratio decreased slightly in both WT and UCP2 mice at 72h. Mitochondrial fission/fusion index in UCP2^-/-^ group was significantly higher than that of the WT group at 24h of reperfusion. These indicated that deletion of UCP2 increased mitochondrial fission/fusion index, tuning the mitochondrial dynamic towards fission. Thus, mitochondrial fission/fusion index was higher in UCP2^-/-^ as compared to WT animals (Fig. 7).

**Figure 8:**
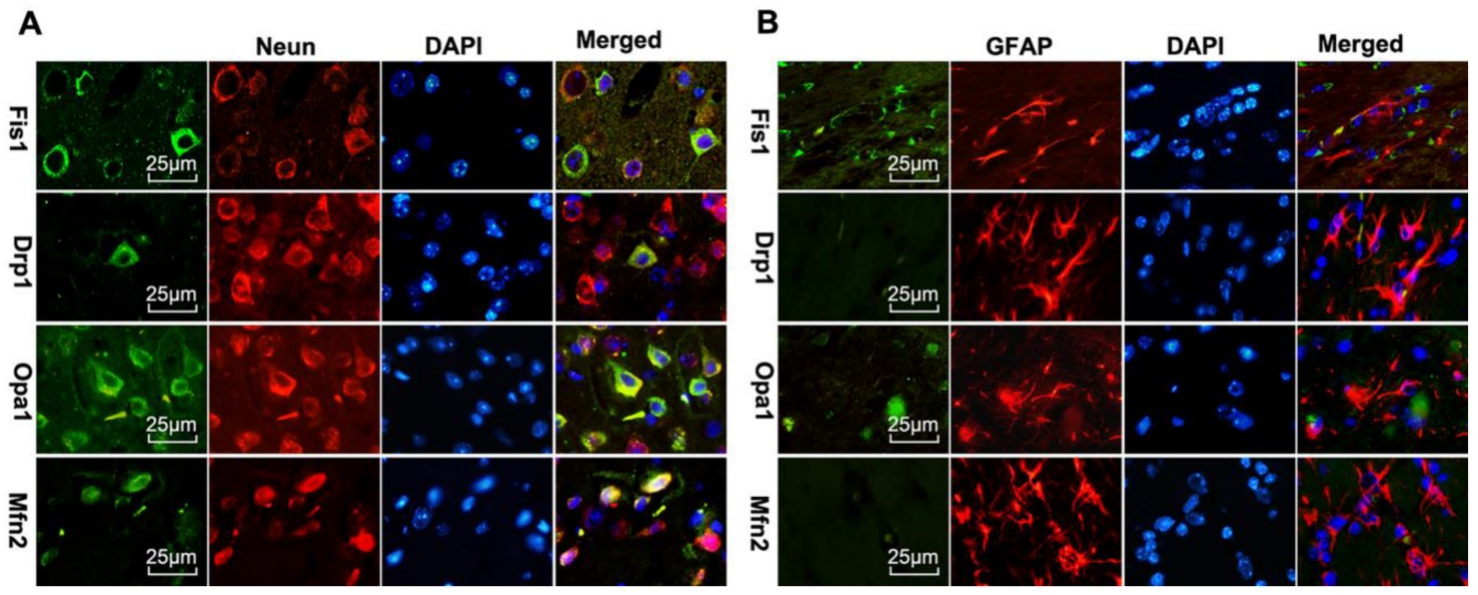
** Mitochondrial fusion-related proteins (Opa1 and Mfn2) and fission-related proteins (Drp1, Fis1) co-localized with neurons in UCP2^-/-^ mice following ischemic stroke.** Double immunostaining of Drp1, Fis1, Opa1 and Mfn2 with GFAP (astrocyte marker) and NeuN (neuron marker) were performed in UCP2^-/-^ mice brain sections 1 day after reperfusion (n=6, each group). Scale bar =25 μm.

Double labeling of the above fission and fusion markers with neuronal marker NeuN (Fig 8A) or astrocyte marker GFAP (Fig 8B) on brain sections 1 day after reperfusion revealed that Drp1, Fis1, Opa1, and Mfn2 were co-localized with NeuN positive neurons, but not with GFAP positive astrocytes, suggesting that mitochondrial fission and fusion occurred majorly in neurons rather than astrocytes.

### Mitochondrial ultrastructural alterations

**Figure 9:**
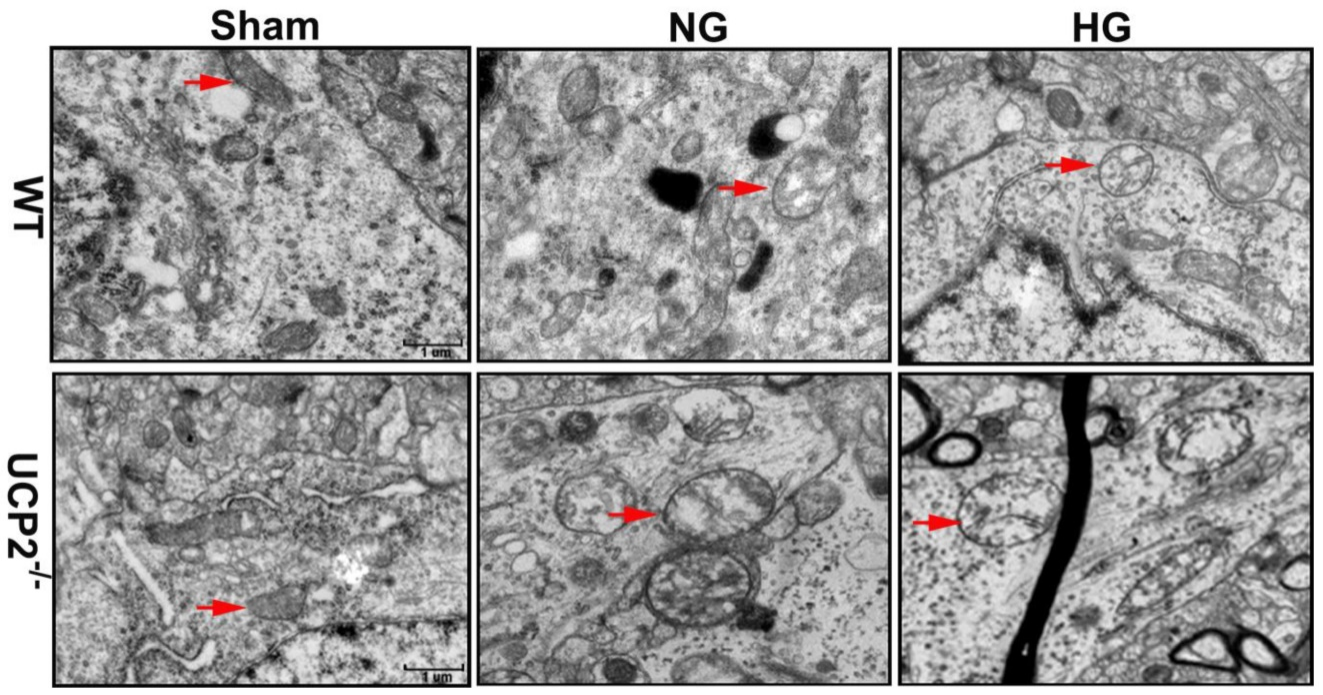
** Mitochondrial ultrastructural alterations.** Representative transmission electron microscope of neuron form cortical area of the brains in UCP2^-/-^ and WT mice after 6h of reperfusion (n=4, each group). Arrow indicates varying mitochondrial size whereas arrowhead shows mitochondrial swelling and disarrayed cristae. Scale bar =1 μm.

To further demarcate the effect of UCP2 on mitochondria, we performed transmission electron microscope to analyze the mitochondrial ultrastructural changes. Mitochondrial morphology was normal in both WT and UCP2^-/-^ non-ischemic control mice. Mild mitochondrial swollen, as reflected by mitochondrial lucency and cristae disarray, was observed in both normoglycemic and hyperglycemic animals after 6h of recovery (Fig. 9, red arrows in WT+NG and WT+HG images). In contrast, deletion of UCP2 resulted in markedly mitochondrial swollen, as reflected by increased mitochondrial size, prominent lucency and cristae disarrangement, in both normo- and hyperglycemic ischemic animals after 6h of reperfusion (Fig. 9, red arrows in UCP2^-/-^+NG and UCP2^-/-^+HG images). These results indicate that homozygous deletion of UCPs gene aggravates mitochondrial structural damage after ischemia and reperfusion in the brain.

## Discussion

Our data demonstrated that the genetic ablation of UCP2 significantly increased infarct volume and brain edema and caused more severe neurological deficit scores after ischemia/reperfusion in both normo- and hyperglycemic animals compared with WT mice subjected to the identical length of ischemia and reperfusion. Further, UCP2 deletion enhanced ROS production in both normo- and hyperglycemic ischemic mice. Moreover, deletion of UCP2 altered mitochondrial dynamic balance by tilting the balance towards fission, especially in hyperglycemic animals after being subjected to ischemia and reperfusion. Finally, deletion of UCP2 resulted in severe mitochondrial ultrastructural damage that is displayed as increases of mitochondrial size and lucency and disarray of mitochondrial cristae.

UCP2 is an inner mitochondrial membrane protein that dissipates the mitochondrial proton gradient by transporting H^+^ across the inner membrane, generating heat, stabilizing the inner mitochondrial membrane potential and reducing the formation of reactive oxygen species (ROS) 23. UCP2 is widely expressed in various tissues including the central nervous system and implicated in diverse pathologic conditions such as obesity, diabetes, neurodegenerative diseases, atherosclerosis, and cancer 24, 25, 26. Emerging evidence suggests that UCP2 may play an important role in cerebral stroke by regulating mitochondrial potential and energy balance, neuroendocrine and autonomic functions, reactive oxygen species (ROS) production and fatty acid anion transport, cell death, and inflammation 27. Among the published literature, a majority of studies have demonstrated that increasing UCP2 is neuroprotective 28. Upregulation of UCP2 has been reported to reduce neuronal damage in cerebral stroke, traumatic brain injury, epilepsy and Parkinson's models 6, 7, 29, 30. Our data demonstrated that the genetic ablation of UCP2 significantly increased infarct volume, brain edema, apoptosis and aggravated neurological deficit scores after ischemia/reperfusion under normoglycemic condition, which is consistent with our previous report and those published in the literature 6, 31, 32. Furthermore, our results for the first time demonstrated that deletion of UCP2 further worsened the ischemic brain damage in hyperglycemic animals comparing to wildtype animals subjected to hyperglycemic ischemia. These results suggest that UCP2 plays an important role in cerebral ischemic injury under both normo- and hyperglycemic conditions.

Reactive oxygen species (ROS) are free radicals that can damage DNA, lipids, and proteins. Hyperglycemia has been found to lead to oxidative stress and increased ROS production in neuronal cells 33, 34. UCP2 reduces the ROS formation by preventing mitochondrial membrane hyperpolarization that in turn inhibits mitochondrial electron transport chain. Studies have shown that UCP2 confers protective effects on various stressors by decreasing mitochondrial ROS production in the brain and liver 35, 36, 37. It has also been reported that UCP2 could protect cardiomyocytes from exogenous oxidant stress 38. In the present study, superoxides were increased after ischemia in normoglycemic animals and further elevated by hyperglycemia. Deletion of UCP2 led to exacerbated levels of ROS after ischemia in both normo- and hyperglycemic animals compared to the counterparts in wildtype animals. These data imply that the increased damage observed in UCP2^-/-^ mice is partially due to increases in ROS production.

Recent studies have suggested that impaired mitochondrial dynamics and excessive mitochondrial fission are connected to several neurodegenerative disorders such as stroke 39, Parkinson's diseases 40, and Alzheimer's diseases 41. In the present study, we also found in both immunohistochemistry and Western blotting that UCP2 deletion caused mitochondrial dynamic imbalance after cerebral ischemic injury under both normo- and hyperglycemic conditions. The results demonstrated that ischemia and reperfusion increased the protein levels of fission proteins Fis1 and Drp1, and decreased fusion proteins Opa1and Mfn2, thereby, tilting the mitochondrial dynamic balance towards fission. Preischemic hyperglycemia further augmented the alteration as reflected by the higher levels of Fis1 and Drp1 in hyperglycemic animals at 6h of reperfusion than those observed in the normoglycemic counterparts, suggesting that hyperglycemia caused an early onset of mitochondrial fission process by hyperglycemia. This is consistent with our previous finding that hyperglycemic ischemia increases mitochondrial dynamic imbalance towards fission 21. When comparing to wildtype animals, deletion of UCP2 further increased the levels of Fis1 and Drp1 in both normo- and hyperglycemic ischemic animals. Previous study shows UCP2 in the ventromedial nucleus of the hypothalamus is required for glucose-induced DRP1-mediated mitochondrial fission 42. However, the impact of UCP2 deletion on mitochondrial fission and fusion balance after cerebral ischemia has not been reported before. A recent study using acute kidney ischemia and reperfusion model demonstrated that UCP knockout mice had pronounced mitochondrial dynamic imbalance towards fission and mitochondrial fragmentation; whereas upregulation of UCP2 ameliorated hypoxia-induced mitochondrial fusion and fission imbalance 43. The immunoreactivity of Opa1 was not changed after ischemia in both normo- and hyperglycemic animals after ischemia and reperfusion up to 72h. That of the Mfn2 decreased in both normo- and hyperglycemic animals with no significant difference between the two glycemic conditions. In contrast, deletion of UCP2 significantly decreased the immunoreactivity of Opa1 and Mfn2 in both normo- and hyperglycemic ischemic animals, suggesting deletion of UCP2 inhibited mitochondrial fusion. Semi-quantitative measurements of Opa1 and Mfn2 by protein blotting revealed marked decreases of these two fusion proteins after ischemia in normoglycemic animals and the decreases were more pronounced in hyperglycemic ischemic animals than in normoglycemic animals, suggesting hyperglycemia inhibited mitochondrial fusion. Deletion of UCP2 further decreased the levels of Opa1 and Mfn2 and the decreases were more obvious in normoglycemic than hyperglycemic animals. The discrepant result of Opa1 between immunohistochemistry and Western blotting may ascribe to the fact that IHC detects the target proteins in specific areas, while ischemic lateral brain was used in Western blotting, which more accurately reflects the changes of fusion protein levels.

Because the general tendency was that ischemia increased fission and decreased fusion proteins, that hyperglycemia further aggravated these alterations, and that UCP2 deletion made the changes even more obvious than in wildtype counterpart animals, we decided to calculate mitochondrial fission/fusion index, which is the ratio of fission proteins divided by fusion proteins. The results clearly demonstrated that ischemia in normoglycemic animals induced mitochondrial fission and hyperglycemia induced a more dramatic increase. These results are further supported by previous publications showing that hyperglycemia causes mitochondrial fission 44, 45. Deletion of UCP2 further increased the fission/fusion index in both normo-and hyperglycemic animals. These data suggest that hyperglycemic ischemia enhances mitochondrial fission after cerebral ischemia and reperfusion and UCP2 deletion further increases mitochondrial dynamic imbalance under both normo- and hyperglycemic conditions. The slanting of the mitochondrial dynamics to fission may reduce the mitochondrial network and enhance mitochondrial rupture and induce neuronal cell death after cerebral ischemia 46. UCP2 silencing has been shown to cause mitochondrial dysfunction in astrocytes under septic conditions 47. However, it is not known whether UCP2 deletion affects the mitochondrial dynamics in astrocytes. Based on our double immunostaining results, the fission and fusion protein markers were co-localized with neuronal marker NeuN, but not with astrocytes. This indicated that UCP2 deficiency mainly affects mitochondrial division and fusion balance in neurons.

Mitochondrial dynamic imbalance could result in mitochondrial morphological alterations 48. Observation of the mitochondrial ultrastructure using an electron microscope found mild to moderately swollen mitochondria with increased lucency and cristae disarrangement in both normo- and hyperglycemic animals as early as 6 h of reperfusion after 1 h of ischemia. In UCP2^-/-^ mice, mitochondrial sizes increased significantly due to swelling. Pronounced lucency and disarray and disappearance of mitochondrial cristae were observed, suggesting that UCP2 deletion increases mitochondrial morphological damage caused by cerebral ischemia in both normo- and hyperglycemic animals. We have previously shown that hyperglycemia aggravates ischemic brain damage 49, 50. This effect is associated with increases of mitochondrial fission and mitochondrial morphological alterations 21, 33, 51. In this study, we observed mild mitochondrial damage including lucency and disarray of cristae in both normo- and hyperglycemic animals at 6 h of reperfusion. UCP2 knockout further aggravated the mitochondrial damage in both normo- and hyperglycemic mice after ischemia and reperfusion. Therefore, mitochondrial size was further enlarged and mitochondrial lucency and vacuolization were more prominent than those being observed in wildtype mice. The impact of UCP2 on mitochondrial morphology of the brain tissue after cerebral ischemia and reperfusion has not been reported. Several studies have demonstrated that silencing UCP2 by small interference RNA resulted in more severe mitochondrial swelling, vacuolization and loss of matrix content in cardiomyocytic H9C2 cells and astrocytes compared with control cells challenged with sepsis 47, 52, 53. These results support our finding that UCP2 plays an important role in maintaining mitochondrial dynamic and morphological integrity in the brain.

Collectively, deletion of UCP2 has an adverse impact on cerebral ischemia-reperfusion injury under both normo- and hyperglycemic conditions. Deletion of UCP2 increases ROS production, mitochondrial fission and morphological abnormalities. These findings may pave the way to new treatment modalities, which are needed for the treatment of brain I/R injury.

## Figures and Tables

**Table 1:** Summary of group and subgroups

Groups	Processing	TTC	Histo	WB	TEM
**WT**					
**NG**					
Sham	Sham-operated	4	10	4	4
6h I/R	MCAO + reperfusion 6h	0	6	6	4
24h I/R	MCAO + reperfusion 24h	4	10	6	0
72h I/R	MCAO + reperfusion 72h	0	6	6	0
**HG**					
Sham	Sham-operated	0	10	4	4
6h I/R	MCAO + reperfusion 6h	0	6	6	4
24h I/R	MCAO + reperfusion 24h	4	10	6	0
72h I/R	MCAO + reperfusion 72h	0	6	6	0
**UCP2^-/-^**					
**NG**					
Sham	Sham-operated	5	10	4	4
6h I/R	MCAO + reperfusion 6h	0	6	6	4
24h I/R	MCAO + reperfusion 24h	5	10	6	0
72h I/R	MCAO + reperfusion 72h	0	6	6	0
**HG**					
Sham	Sham-operated	0	10	4	4
6h I/R	MCAO + reperfusion 6h	0	6	6	4
24h I/R	MCAO + reperfusion 24h	5	10	6	0
72h I/R	MCAO + reperfusion 72h	0	6	6	0

WT: Wildtype mice; UCP2^-/-^: knock out UCP2 mice; NG: normoglycemia; HG: hyperglycemia; 6h I/R, 24h I/R and 72h I/R: MCAO and reperfusion 6h, 24h, and 72h. MCAO: middle cerebral artery occlusion; TTC: 2,3,5-triphenyltetrazolium chloride; Histo: histology; WB: Western blot; TEM: transmission electron microscopy.

## Abbreviations

UCPs: Uncoupling proteins
UCP2: Uncoupling protein 2
UCP2^-/-^: UCP2 deletion
I/R: ischemia/reperfusion
MCAO: middle cerebral artery occlusion
WT: wildtype
NG: normoglycemic
HG: hyperglycemic
TUNEL: Terminal deoxynucleotidyl transferase mediated dUTP nick‑end labeling
ROS: reactive oxygen species
Fis1: fission 1
Drp1: dynamin-related protein 1
Opa1: optic atrophy 1
Mfn2: mitofusin 2
STZ: Streptozotocin
TTC: 2,3,5-Triphenyl Tetrazolium Chloride
ICA: internal carotid artery
ECA: external carotid artery
CCA: common carotid artery
DHE: Dihydroethidium
